# High expression of UNC5B enhances tumor proliferation, increases metastasis, and worsens prognosis in breast cancer

**DOI:** 10.18632/aging.103639

**Published:** 2020-09-09

**Authors:** Shijie Wu, Xinyue Guo, Jiaojiao Zhou, Xuan Zhu, Huihui Chen, Kun Zhang, Yuexin Lu, Yiding Chen

**Affiliations:** 1 Department of Breast Surgery, The Second Affiliated Hospital, Zhejiang University School of Medicine, Hangzhou 310009, Zhejiang, China; 2 The Key Laboratory of Cancer Prevention and Intervention, China National Ministry of Education, Zhejiang University School of Medicine, Hangzhou 310009, Zhejiang, China; 3 Department of Obstetrics and Gynecology, Women’s Hospital, Zhejiang University School of Medicine, Hangzhou 310009, Zhejiang, China; 4 Department of Radiation Oncology, The First Affiliated Hospital, Zhejiang University School of Medicine, Hangzhou 310009, Zhejiang, China

**Keywords:** UNC5B, breast cancer, prognosis, metastasis, functional networks

## Abstract

UNC-5 Homolog B (UNC5B) is a member of the dependence receptor family that regulates cell survival and apoptosis in a ligand-dependent manner. UNC5B plays an important role in the development of multiple cancers, including colorectal, bladder, and thyroid cancer. However, the exact expression pattern and mechanism of UNC5B in breast cancer have not been well elucidated. Here, we showed that UNC5B expression was significantly upregulated in breast cancer using bioinformatics analysis and experimental validation. High UNC5B expression was correlated with poor overall survival in breast cancer patients. UNC5B knockdown inhibited breast cancer cell proliferation and metastasis and compromised PI3K/Akt signaling activation. In summary, UNC5B is a promising diagnostic and prognostic biomarker and targeting UNC5B is a potential strategy for individualized breast cancer treatment.

## INTRODUCTION

Worldwide, breast cancer is the most commonly diagnosed cancer and the leading cause of cancer death in women [1]. In the last few decades, the treatment efficacy of breast cancer has improved tremendously, and various prognostic biomarkers such as estrogen receptor (ER), progesterone receptor (PR), and human epidermal growth factor receptor 2 (HER2) have been identified [2, 3]. With the help of these molecular biomarkers, endocrine therapy such as tamoxifen and targeted therapy such as trastuzumab have been used as first-line options for ER-positive and HER2-positive breast cancer patients, respectively [4, 5]. Despite the great success of early screening and biomarker-based therapies in breast cancer treatment, some patients already have distant metastases when diagnosed with breast cancer and some patients acquire resistance to therapies such as tamoxifen [6], which severely impairs their prognosis [7]. Therefore, effective diagnostic biomarkers and new therapeutic targets for breast cancer are urgently needed.

UNC-5 Homolog B (UNC5B), first described as an axon-guiding transmembrane receptor, is a member of the human UNC5 homologs (UNC5H) comprising UNC5A, UNC5B, UNC5C, and UNC5D [8]. UNC5B plays crucial roles in multiple biological processes, including regulating vascular morphogenesis and acting as a repellent in axonal migration [9, 10]. In addition, UNC5B belongs to the dependence receptor family, which is defined by common functional characteristics and initiates two distinct signaling pathways depending on the presence or absence of ligand [11]. UNC5B can trigger apoptosis by activating death-associated protein kinase (DAPK) in the absence of ligand [12], whereas its proapoptotic effect is blocked in the presence of netrin-1 [13], a survival factor in tumorigenesis [14–17]. Because of its well-known effects on apoptosis and cell survival, UNC5B is implicated in tumorigenesis. UNC5B expression is dysregulated in many cancers, including colorectal, bladder, and thyroid cancer [18–20] and UNC5B knockdown suppresses the proliferation, migration, and invasion of thyroid cancer cells [20]. Nevertheless, the expression level of UNC5B and its clinical relevance in breast cancer have not been fully elucidated. Moreover, the molecular function and underlying mechanism of UNC5B in breast cancer are unclear.

In this study, we showed that UNC5B was frequently overexpressed in breast cancer. High UNC5B expression was correlated with poor prognosis in breast cancer patients. We also noted that UNC5B inhibition attenuated the proliferation and metastasis of breast cancer cells and demonstrated that UNC5B depletion inhibited PI3K/Akt signaling. Our findings highlight the significance of UNC5B in breast cancer, including its promising diagnostic and prognostic value and potential as a therapeutic target.

## RESULTS

### UNC5B is predominant in breast cancer

**Figure 1:**
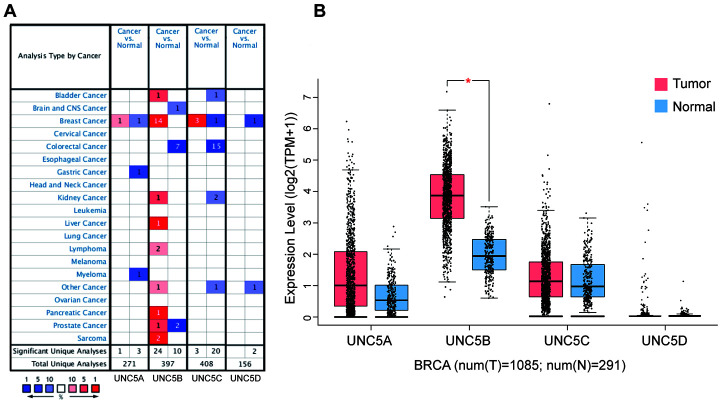
**UNC5B is overexpressed in breast cancer (ONCOMINE and GEPIA).** (**A**) Different mRNA expression of UNC5H receptors between cancer tissues and normal tissues. (**B**) mRNA expression levels of UNC5H receptors in breast cancer tissues and normal breast tissues. *, *p* < 0.05.

Based on the ONCOMINE database (http://www.oncomine.org), we first analyzed the mRNA expression of UNC5H receptors in multiple cancers and normal tissues (Figure 1A). Compared with normal breast tissues, UNC5B mRNA expression was significantly upregulated in breast cancer tissues in 14 datasets (*p* < 0.05) (Table 1). Analysis of the breast cancer dataset available through The Cancer Genome Atlas (TCGA) and GTEx database confirmed a marked increase in UNC5B expression in breast cancer compared to normal breast tissues (*p* < 0.05), whereas there were no significant differences in UNC5A, UNC5C, and UNC5D expression between breast cancer and normal tissues (Figure 1B). Thus, further analyses focused on UNC5B. We further analyzed the UNC5B expression in 32 cancer types utilizing the TCGA database. UNC5B mRNA expression was consistently higher in breast cancer tumors than in adjacent normal tissues (*p* < 0.001) (Supplementary Figure 1A). Meanwhile, UNC5B expression was significantly higher in cholangiocarcinoma, kidney renal clear cell carcinoma, and thyroid carcinoma compared with adjacent normal tissues (*p* < 0.001) (Supplementary Figure 1A). We subsequently analyzed UNC5B expression in three major histological subtypes of breast cancer, including invasive ductal breast carcinoma, lobular breast carcinoma, and mixed lobular and ductal breast carcinoma. Consistently, UNC5B expression was higher in tumors than in adjacent normal tissues among these three breast cancer subtypes (Supplementary Figure 1B–1I).

**Table 1:** Significant changes in UNC5B expression at the transcriptional level between different histological subtypes of breast cancer and normal breast tissues (ONCOMINE).

**Types of breast cancer VS. normal breast tissue**	**Fold change**	***p* value**	**t-test**	**Overexpression gene rank**	**Reference**
Invasive Ductal Breast Carcinoma	4.337	3.53E-6	5.772	top 1%	Turashvili Breast [43]
Invasive Lobular Breast Carcinoma	2.382	0.046	1.938	top 9%	Turashvili Breast [43]
Ductal Breast Carcinoma in Situ Stroma	4.726	1.18E-8	8.285	top 1%	Ma Breast 4 [44]
Invasive Ductal Breast Carcinoma Stroma	4.731	4.45E-6	6.394	top 1%	Ma Breast 4 [44]
Invasive Breast Carcinoma	3.318	8.58E-25	12.692	top 2%	TCGA Breast^a^
Invasive Ductal Breast Carcinoma	3.512	8.39E-38	19.232	top 2%	TCGA Breast^a^
Invasive Lobular Breast Carcinoma	4.147	2.97E-19	12.105	top 1%	TCGA Breast^a^
Mixed Lobular and Ductal Breast Carcinoma	5.947	1.45E-4	6.706	top 5%	TCGA Breast^a^
Male Breast Carcinoma	6.661	1.32E-10	25.621	top 1%	TCGA Breast^a^
Intraductal Cribriform Breast Adenocarcinoma	7.027	2.41E-6	21.314	top 2%	TCGA Breast^a^
Invasive Ductal Breast Carcinoma	3.705	8.00E-7	10.125	top 5%	Zhao Breast [45]
Lobular Breast Carcinoma	2.827	9.18E-6	8.016	top 4%	Zhao Breast [45]
Invasive Ductal Breast Carcinoma Stroma	3.741	0.001	4.155	top 4%	Karnoub Breast [46]
Invasive Breast Carcinoma	3.456	0.002	6.470	top 9%	Gluck Breast [47]

^a^TCGA: The Cancer Genome Atlas.

**Figure 2:**
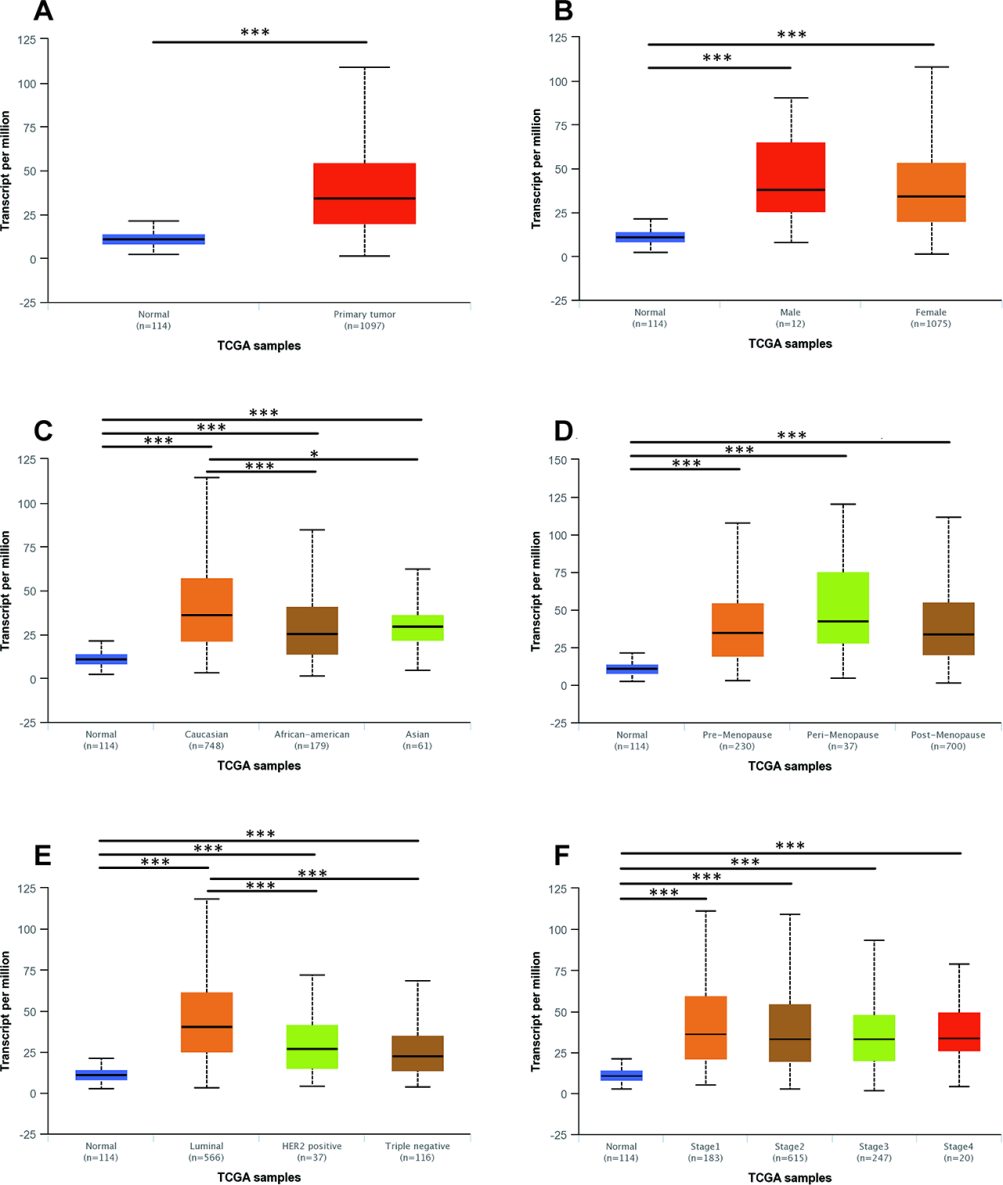
**The mRNA expression of UNC5B in breast cancer patients with distinct clinicopathological features (UALCAN).** (**A**) UNC5B mRNA expression in normal breast tissues and breast cancer tissues. (**B**) UNC5B mRNA expression in normal breast tissues and breast cancer tissues classified by gender. (**C**) UNC5B mRNA expression in normal breast tissues and breast cancer tissues classified by ethnicity. (**D**) UNC5B mRNA expression in normal breast tissues and breast cancer tissues classified by menopause status. (**E**) UNC5B mRNA expression in normal breast tissues and breast cancer tissues classified by molecular subtype. (**F**) UNC5B mRNA expression in normal breast tissues and breast cancer tissues classified by cancer stage. *, *p* < 0.05; **, *p* < 0.01; ***, *p* < 0.001.

We then evaluated correlations between UNC5B expression and clinicopathological features in breast cancer patients. A total of 1,097 primary breast tumor samples and 114 normal breast tissues were analyzed in UALCAN (http://ualcan.path.uab.edu). Across gender, race, menopause status, molecular subtypes, and cancer stages, UNC5B mRNA expression was always higher in breast cancer patients than in healthy individuals (Figure 2). Note that white breast cancer patients tended to have higher UNC5B expression than African-American patients (*p* < 0.001) and Asian patients (*p* < 0.05), implying ethnic diversity of UNC5B expression (Figure 2C). In addition, the luminal subtype expressed UNC5B at a higher level than the HER2-positive (*p* < 0.001) and triple-negative subtypes (*p* < 0.001) (Figure 2E).

### UNC5B protein expression in breast cancer

**Figure 3:**
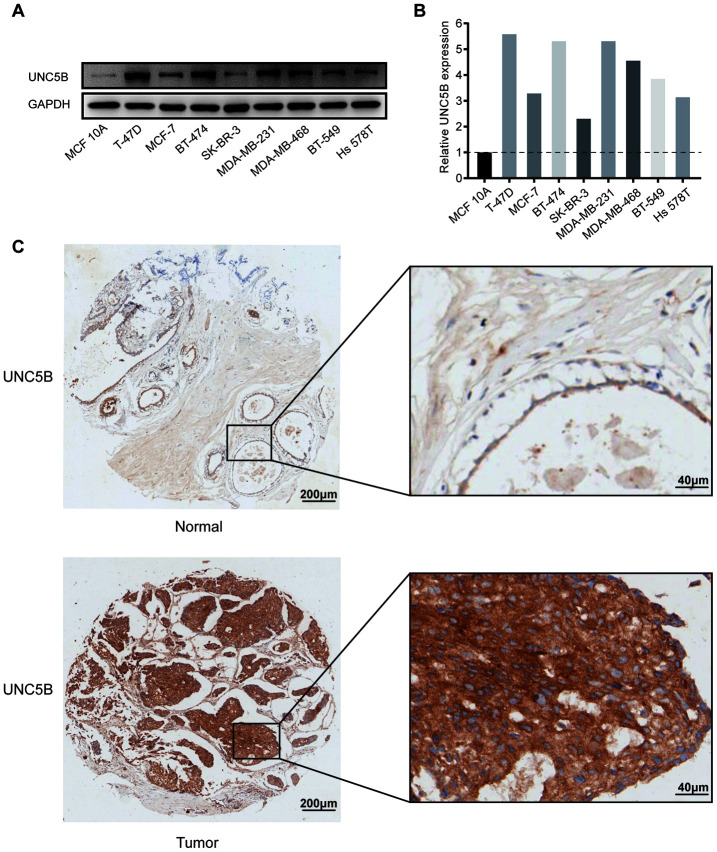
**UNC5B protein expression in breast cancer (Western blot and IHC).** (**A**) UNC5B expression in MCF 10A cells and diverse breast cancer cell lines. (**B**) The relative UNC5B protein expression in MCF 10A cells and diverse breast cancer cell lines. (**C**) Representative IHC image of UNC5B expression in breast cancer tissues and normal breast tissues.

We further evaluated UNC5B protein expression in diverse breast cancer cell lines and MCF 10A cells, a nontumorigenic breast epithelial cell line, by performing Western blot. As expected, UNC5B expression was upregulated in eight breast cancer cell lines compared to MCF 10A cells (Figure 3A and 3B).

In addition, we analyzed UNC5B expression in breast cancer tissues and normal breast tissues using immunohistochemistry (IHC). UNC5B was expressed at a higher level in breast cancer tissues than in normal tissues (Figure 3C). Considering the close association of UNC5B with breast cancer, UNC5B could act as a potential diagnostic marker of breast cancer.

### High UNC5B expression associated with poor prognosis in breast cancer patients

**Figure 4:**
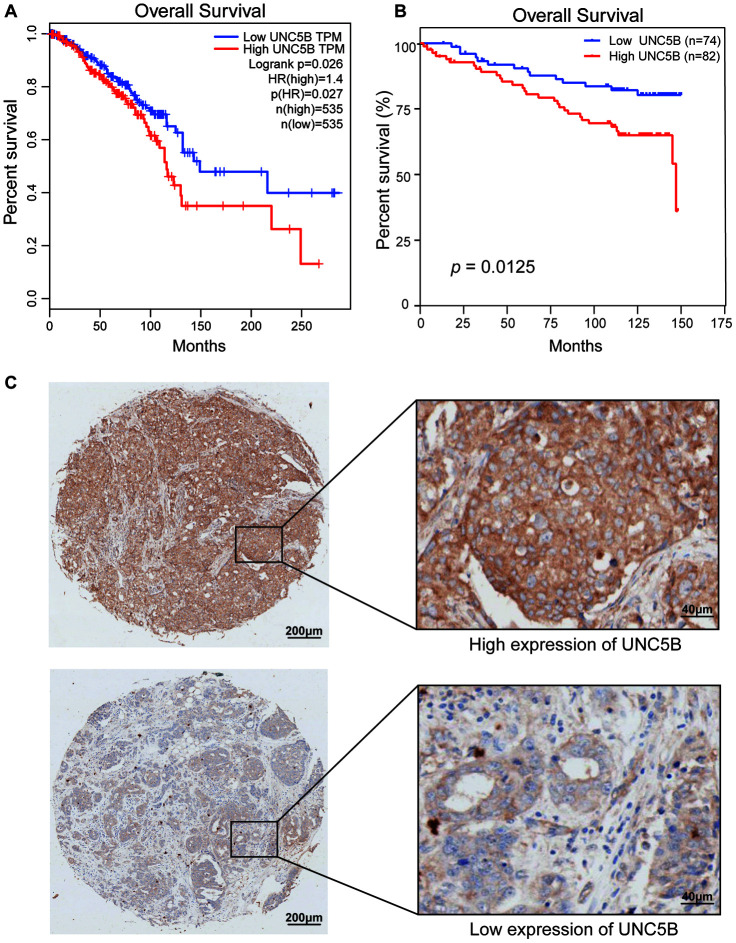
**The prognostic value of UNC5B expression in breast cancer (GEPIA and IHC).** (**A**) The prognostic value of UNC5B mRNA expression in breast cancer patients, analyzed by GEPIA. (**B**) The prognostic value of UNC5B protein expression in breast cancer patients. (**C**) Representative IHC images of high expression and low expression of UNC5B in breast cancer tissues.

We then investigated the prognostic value of UNC5B in breast cancer. Interestingly, survival analysis using GEPIA (http://gepia.cancer-pku.cn/index.html) indicated that increased UNC5B mRNA expression was significantly associated with poor overall survival (OS) in breast cancer patients (*p* = 0.026) (Figure 4A). By using a breast cancer tissue microarray (TMA), we further evaluated the prognostic value of UNC5B at the protein level. The results of the IHC staining revealed that high UNC5B protein expression was correlated with poor OS in breast cancer patients (*p* = 0.0125) (Figure 4B and 4C). These results suggest that UNC5B might be a promising prognostic biomarker in breast cancer treatment.

### Genomic alterations of UNC5B and their biological functions in breast cancer

**Figure 5:**
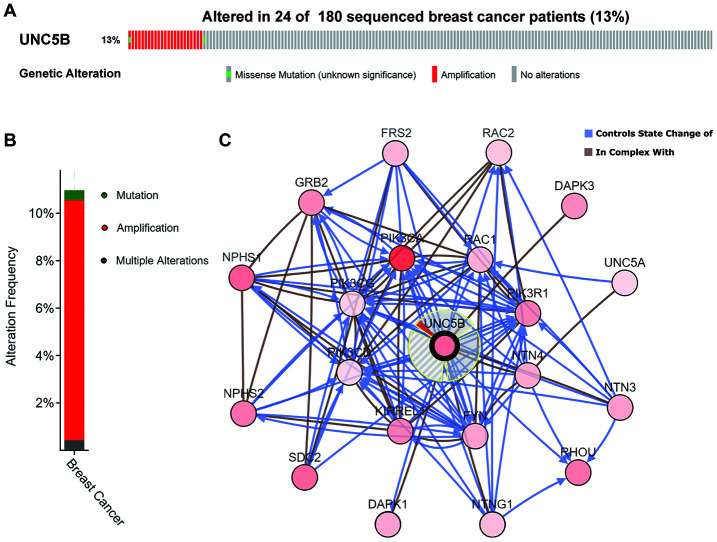
**Genomic alterations of UNC5B and the biological interaction network in breast cancer (cBioPortal).** (**A**, **B**) Types and frequencies of UNC5B alterations in breast cancer. (**C**) The biological interaction network of UNC5B and its neighboring altered genes in breast cancer. UNC5B is represented with a thick border, and the interaction types are shown in the legend.

We next evaluated genomic alterations of UNC5B in breast cancer using cBioPortal (http://cbioportal.org). UNC5B was altered in 24 of 180 (13%) breast cancer patients (Figure 5A), and amplification was the most frequent type of UNC5B alteration in breast cancer (10.13%) (Figure 5B). The *Network* model of cBioPortal was used to generate an interaction network of the 20 most frequently altered UNC5B-neighboring genes. The results showed that cancer-related genes, including PIK3CA, PIK3CD, PIK3R1, DAPK1, DAPK3, RAC1, RAC2, and GRB2, were associated with UNC5B alterations (Figure 5C). Detailed characteristics of UNC5B-neighboring gene alterations are shown in Supplementary Table 1.

**Figure 6:**
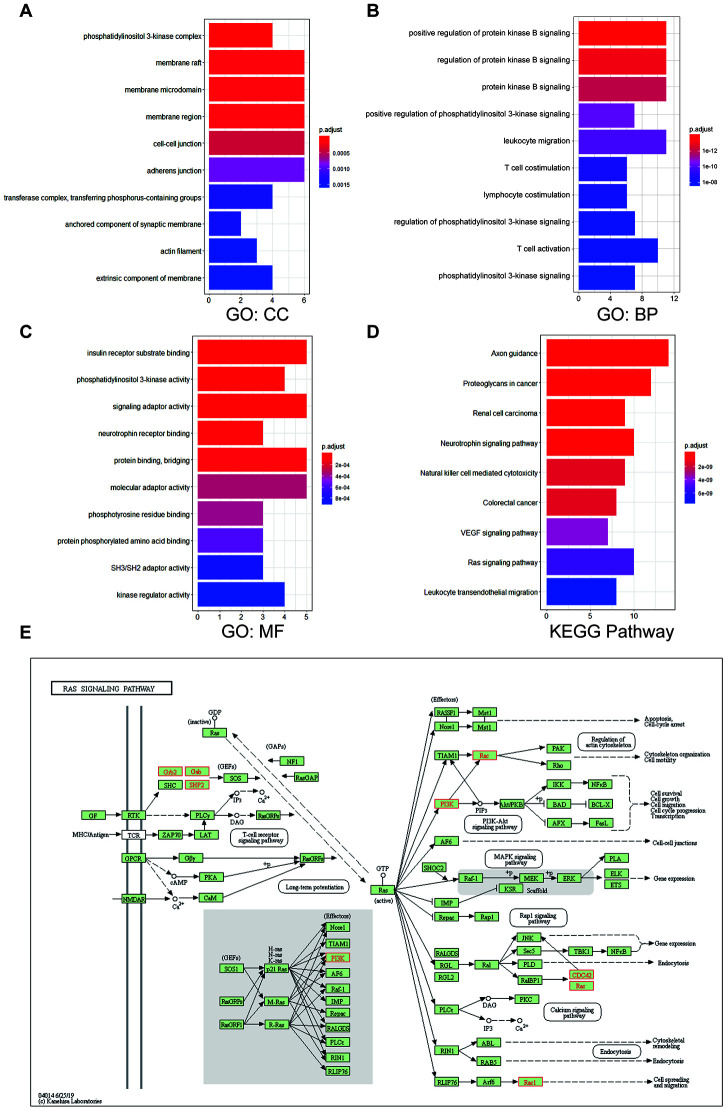
**Enrichment analysis of UNC5B and its neighboring altered genes in breast cancer.** The functions of UNC5B and the 20 most frequently altered UNC5B-neighboring genes were predicted in Gene Ontology (GO) and Kyoto Encyclopedia of Genes and Genomes (KEGG) pathway enrichment analyses using the “clusterProfiler” package in R. (**A**) Cellular components. (**B**) Biological processes. (**C**) Molecular functions. (**D**) KEGG pathway enrichment analysis. (**E**) Detailed annotation of the Ras signaling pathway regulated by UNC5B-associated gene alterations. Nodes marked in red represent altered genes.

To further investigate the functions of UNC5B and the 20 most frequently altered UNC5B-neighboring genes, we performed Gene Ontology (GO) and Kyoto Encyclopedia of Genes and Genomes (KEGG) pathway enrichment analyses using the “clusterProfiler” package in R [21]. These genes were primarily involved in the composition of the phosphatidylinositol 3-kinase (PI3K) complex, membrane structures, and cell-cell junctions (Figure 6A). Similarly, these gene alterations primarily affected the regulation of PI3K signaling and protein kinase B signaling (Figure 6B). Moreover, these gene alterations significantly affected molecular functions, such as PI3K activity, protein binding, and signaling adaptor activity (Figure 6C). In the KEGG pathway analysis, the results showed obvious enrichments in cancer-associated signaling pathways, including the Ras signaling pathway (Figure 6D and 6E).

### Knockdown of UNC5B in breast cancer cells inhibited their proliferation and metastasis

**Figure 7:**
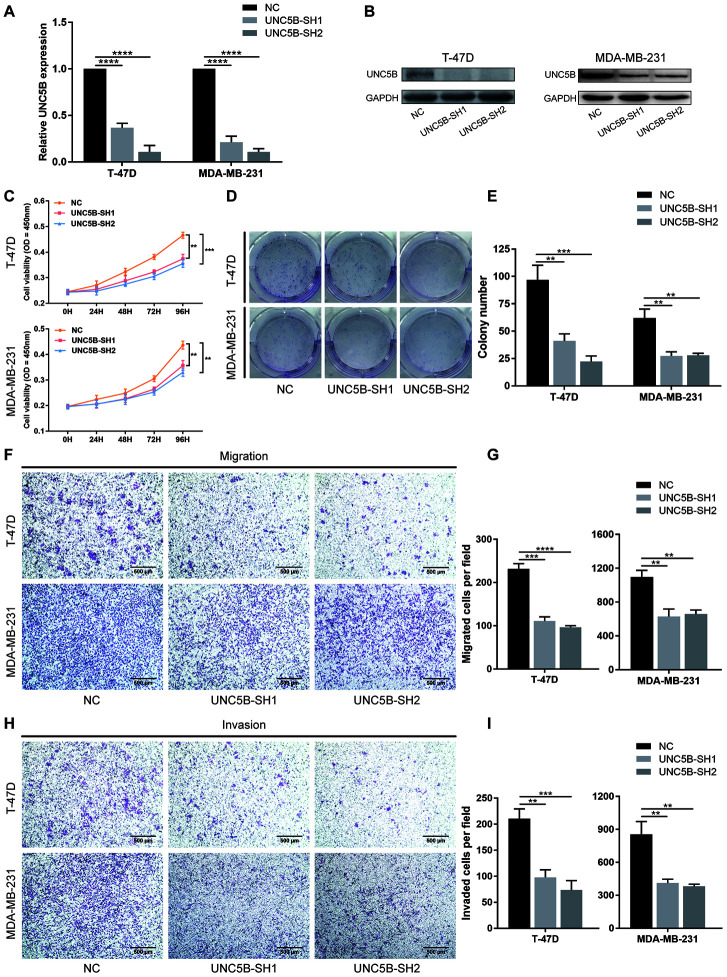
**UNC5B knockdown inhibited the proliferation and metastatic capacities of breast cancer cells.** (**A**) Quantitative PCR analysis of UNC5B expression in T-47D and MDA-MB-231 cells after UNC5B-shRNA introduction. (**B**) Western blot analysis of UNC5B expression in T-47D and MDA-MB-231 cells after UNC5B-shRNA introduction. (**C**) The proliferation of T-47D and MDA-MB-231 cells after UNC5B knockdown. (**D**, **E**) Representative images and quantified results of colonies formation in T-47D and MDA-MB-231 cells after UNC5B knockdown. (**F**, **G**) Representative images and quantified results of transwell migration assay in T-47D and MDA-MB-231 cells after UNC5B knockdown. (**H**, **I**) Representative images and quantified results of matrigel-treated transwell invasion assay in T-47D and MDA-MB-231 cells after UNC5B knockdown. Data represent three independent experiments. Data are expressed as mean ± s.e.m. *p < 0.05, **p < 0.01, ***p < 0.001, ****p < 0.0001.

To elucidate the specific role of UNC5B in breast cancer, we performed shRNA-mediated UNC5B knockdown in T-47D and MDA-MB-231 breast cancer cells. Quantitative RT-PCR revealed that UNC5B mRNA expression was significantly inhibited by UNC5B-shRNA (UNC5B-SH1 and UNC5B-SH2) (*p* < 0.0001) (Figure 7A). Western blot analysis confirmed a marked decrease in UNC5B expression in UNC5B-shRNA treated breast cancer cells compared to the negative control (NC)-shRNA treated cells (Figure 7B). As expected, UNC5B depletion significantly attenuated the proliferation of breast cancer cells (*p* < 0.01) (Figure 7C). Consistently, the silencing of UNC5B compromised the colony formation ability of breast cancer cells (Figure 7D and 7E). We further investigated the effect of UNC5B on breast cancer metastasis. As shown in Figure 7F–7I, the silencing of UNC5B significantly suppressed the migration and invasion capacities of breast cancer cells. Collectively, these results emphasized the essential role of UNC5B in breast cancer cell proliferation and metastasis.

### Knockdown of UNC5B in breast cancer cells inhibited PI3K/Akt pathway activation

To further explore UNC5B co-expressed genes in breast cancer, we used the LinkedOmics database (http://linkedomics.org/login.php) to analyze mRNA sequencing data from 1,093 breast cancer patients in TCGA. As shown in the volcano plot (Supplementary Figure 2A), 6,672 genes were significantly positively correlated with UNC5B and 6,161 genes were significantly negatively correlated (false discovery rate (FDR) < 0.05). COL5A1 was the most significantly positively correlated with UNC5B (Pearson correlation = 0.68, *p* = 1.557e-147) (Supplementary Figure 2B). The top 50 genes positively and negatively correlated with UNC5B in breast cancer are shown in heat maps (Supplementary Figure 2C and 2D).

**Figure 8:**
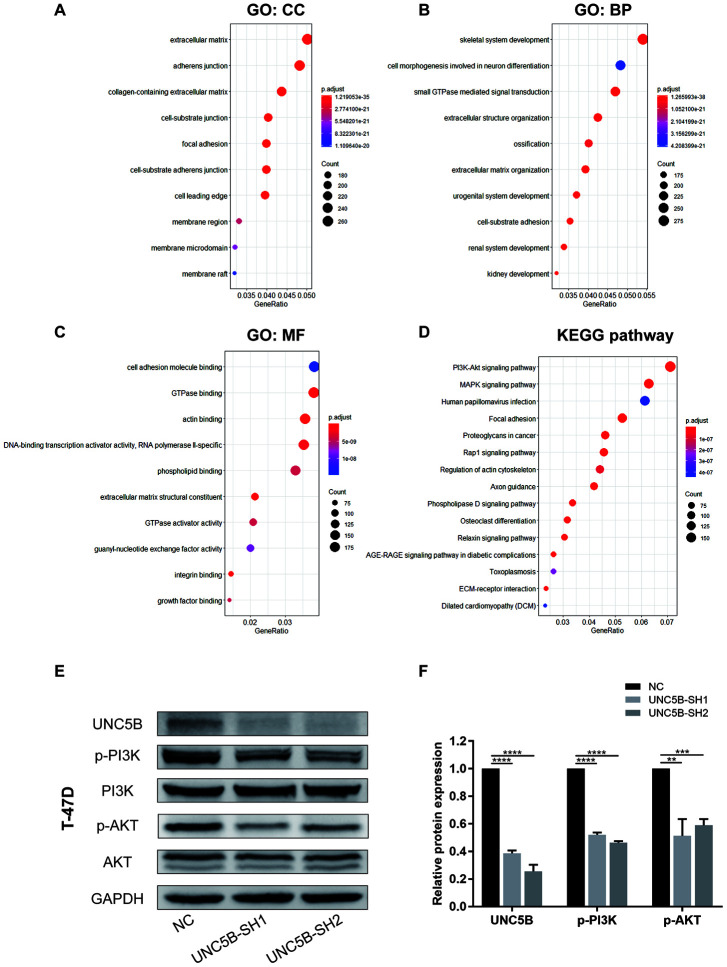
**UNC5B knockdown compromised PI3K/Akt signaling activation in breast cancer cells.** (**A**–**D**) The functions of UNC5B and genes significantly co-expressed with UNC5B were predicted in GO and KEGG pathway enrichment analyses using the “clusterProfiler” package in R. (**A**) Cellular components. (**B**) Biological processes. (**C**) Molecular functions. (**D**) KEGG pathway enrichment analysis. (**E**, **F**) Western blot analysis of PI3K and Akt phosphorylation in breast cancer cells after UNC5B knockdown. Data represent three independent experiments. Data are expressed as mean ± s.e.m. **p < 0.01, ***p < 0.001, ****p < 0.0001.

The functions of these UNC5B co-expressed genes were explored using GO and KEGG pathway enrichment analyses. Proteins from these genes were mainly located in the extracellular matrix, adherens junctions, and membrane regions (Figure 8A). These genes were involved in multiple biological processes, including small GTPase-mediated signaling transduction and extracellular structure organization (Figure 8B). Similarly, these genes remarkably affected the molecular functions of cell adhesion molecule binding and GTPase binding (Figure 8C). Notably, KEGG enrichment analysis indicated widespread changes in biological pathways, among which the PI3K/Akt signaling pathway was prominently enriched (Figure 8D). To demonstrate whether UNC5B regulates the PI3K/Akt signaling pathway in breast cancer, we analyzed PI3K/Akt pathway activation using Western blot analysis. As shown in Figure 8E–8F, the phosphorylation of PI3K and Akt were significantly blunted in UNC5B-knockdown breast cancer cells compared with breast cancer cells treated with NC-shRNA, suggesting the essential role of UNC5B in PI3K/Akt pathway activation.

## DISCUSSION

Metastasis and drug resistance are critical challenges in the clinical management of breast cancer. Despite advances in noninvasive breast surveillance, including breast ultrasound, mammography, and MRI, approximately 4-6% of breast cancer patients have metastases when diagnosed, compromising the effectiveness of subsequent therapies [22]. In addition, drug resistance, such as chemoresistance in triple-negative breast cancer patients, usually leads to tumor progression and poor prognosis [23]. Hence, identifying specific diagnostic biomarkers and novel therapeutic targets would be incredibly valuable in the treatment of breast cancer.

In the present study, we demonstrated for the first time that UNC5B mRNA expression was significantly higher in breast cancer tissues than in normal breast tissues using transcriptional data from more than 1,000 breast cancer samples in diverse databases. Further subgroup analyses confirmed that UNC5B expression was upregulated in breast cancer tissues consistently, regardless of histological subtypes and other clinicopathological features, including gender, race, menopause status, molecular subtype, and cancer stage. Similarly, we verified that UNC5B expression was upregulated in breast cancer cell lines and breast cancer tissues using Western blot and IHC, respectively. These findings suggest UNC5B as a promising diagnostic biomarker in breast cancer. Interestingly, in 2003, Thiebault et al. noted that UNC5B expression was downregulated in breast cancer compared with normal breast tissues, which was contrary to our findings [24]. This conclusion came from the analysis of a dot blot array using a complete rat UNC5H2 cDNA probe that could hybridize UNC5A, UNC5B, and UNC5C cDNA rather than specifically binding to UNC5B. This lack of specificity may be the key difference between this conclusion and ours.

To understand how UNC5B influences breast cancer prognosis, we analyzed the expression of UNC5B and its prognostic value in breast cancer using the TCGA data in GEPIA. The results showed that increased UNC5B expression was significantly correlated with poor OS in breast cancer patients. Protein level validation in a breast cancer TMA confirmed that high UNC5B expression could serve as a biomarker for poor prognosis in breast cancer. Intriguingly, the expression of UNC5H receptors was downregulated in colorectal cancer, and UNC5C loss-of-function was associated with intestinal tumor progression in mice [24, 25]. In this regard, UNC5H receptors were proposed to be tumor suppressors. Nevertheless, netrin-1 expression in the mouse gut reduced intestinal cell apoptosis and facilitated intestinal tumor development [26]. Hence, UNC5H receptors could serve as conditional tumor suppressors depending on the presence of netrin-1, theoretically providing two ways to impact tumorigenesis [27]. First, UNC5H expression could be reduced to block the proapoptotic effect of these receptors in the absence of netrin-1, as occurs in colorectal cancer cells. Second, the antiapoptotic effect of these receptors could be enhanced in the presence of netrin-1. Along this line, UNC5B overexpression in breast cancer as observed in our study could be a selective advantage for tumorigenesis, as increased expression of netrin-1 was seen in the majority of breast tumors with metastatic tendency [14]. In addition, our results showed that UNC5B knockdown significantly inhibited the proliferation and metastasis of breast cancer cells, underscoring the essential role of UNC5B in breast cancer development.

Recent studies have emphasized the importance of genomic alterations in cancer development [28]. The identification of several mutational cancer drivers, including ER, HER-2, PIK3CA, and CDK4/6, promoted the development of stratified medicine in breast cancer [29]. Here, we discovered that UNC5B was altered in 24 of 180 sequenced breast cancer patient samples (13%) and that the major type of UNC5B alteration was amplification. Interestingly, further analysis of the interaction network of UNC5B and its neighboring altered genes indicated that genomic alterations to these genes mainly influenced the activities of key kinases in signaling transduction pathways, including the Ras signaling pathway. Indeed, the Ras signaling pathway plays a vital role in regulating cell survival and abnormal Ras signal transduction promotes cancer development [30]. Thus, UNC5B is a promising target for individualized breast cancer management.

Various studies have demonstrated the importance of the PI3K/Akt signaling pathway in breast cancer [31]. Tumor cells aberrantly activate the PI3K/Akt pathway to enhance cell proliferation, survival, and drug resistance [32]. However, the use of PI3K inhibition in clinical trials showed only a modest effect in breast cancer patients [33, 34], mainly because of drug-related toxicities and compensatory mechanisms [35]. In our study, UNC5B co-expressed genes were significantly enriched in the PI3K/Akt signaling pathway and UNC5B depletion in breast cancer cells markedly inhibited the PI3K/Akt pathway. This was consistent with the previous finding that UNC5B regulated cell survival through PI3K-mediated activation of Akt in response to stresses [36]. Consequently, targeting UNC5B may enhance the clinical application of PI3K inhibitors and improve the prognosis of breast cancer patients.

In summary, our study provides a comprehensive understanding of the role of UNC5B in breast tumorigenesis and better defines the value of UNC5B in breast cancer management. Our data revealed that upregulated UNC5B expression in breast cancer could be a promising diagnostic biomarker. High UNC5B expression and poor OS are correlated, further suggesting UNC5B as a potential prognostic biomarker in breast cancer. Moreover, UNC5B knockdown mitigated the aggressiveness of breast cancer cells and compromised PI3K/Akt pathway activation, suggesting UNC5B as a new therapeutic target for breast cancer. One limitation of our study was that the UNC5B genomic alteration data in breast cancer were obtained from online databases and were not validated in our datasets. In addition, the molecular functions of UNC5B in breast cancer were evaluated *in vitro*. Further work is required to validate the spectrum of UNC5B genomic alterations, and *in vivo* experimental investigations are needed to validate the essential role of UNC5B in breast cancer, which could contribute to the development of precision medicine.

## MATERIALS AND METHODS

### Ethics statement

This study was approved by the Medical Ethics Committee of the Second Affiliated Hospital of Zhejiang University School of Medicine. All datasets were obtained from public databases and all data were collected with written informed consent.

### ONCOMINE database

ONCOMINE database (http://www.oncomine.org) is a comprehensive cancer microarray database and visualized data-mining platform [37]. In our study, the mRNA expression of UNC5H receptors between different types of cancers and adjacent normal tissues were analyzed by the ONCOMINE database. The thresholds used were *p* value of 0.05, fold change of 2, and gene rank of top 10%. UNC5B transcriptional expression in different histological subtypes of breast cancer was also assessed by ONCOMINE, and Student’s t-test was used to generate a *p* value.

### TIMER database

TIMER (https://cistrome.shinyapps.io/timer/) is an integrated resource for dynamic analysis of gene expression profiles and tumor-immune interactions across 32 cancer types [38]. We used the *Dfii ExP* module to determine UNC5B expression of tumor tissues and adjacent normal tissues in 32 cancer types from TCGA.

### UALCAN

UALCAN (http://ualcan.path.uab.edu) is an interactive web interface for in-depth analyses of level 3 RNA-seq and clinical data of 31 cancer types from the TCGA database [39]. We used the *Expression* module to explore the relative UNC5B mRNA expression of invasive breast carcinoma and adjacent normal breast tissues based on different clinicopathologic parameters, including gender, ethnicity, menopause status, molecular subtypes, and cancer stages. Student’s t-test was used to generate a *p* value with *p* < 0.05 considered statistically significant.

### Cell culture

MCF 10A cells and the human breast cancer cell line T-47D were purchased from ATCC (Gaithersburg, Maryland), and other cell lines, including MCF-7, BT-474, SK-BR-3, MDA-MB-231, MDA-MB-468, BT-549, and Hs 578T, were obtained from the Cell Bank of the Chinese Academy of Sciences (Shanghai, China). All cell lines used were analyzed by short tandem repeat profiling, and the culture conditions of these cell lines followed ATCC protocols.

### UNC5B knockdown in breast cancer cells

For stable knockdown of UNC5B, lentivirus stably expressing UNC5B-shRNA or control-shRNA (OBiO, Shanghai, China) was introduced into breast cancer cells. Knockdown efficiency was verified by quantitative RT-PCR and Western blot. The sequences of UNC5B-shRNA and NC-shRNA were as follows. UNC5B-SH1: 5’-CCGGCCACACAGATCTACTTCAATTCAAGAGATTGAAGTAGATCTGTGTGGTTTTTTG-3’; UNC5B-SH2: 5’-CCGGCCAACTTCCTGCTCACCATTTCAAGAGAATGGTGAGCAGGAAGTTGGTTTTTTG-3’; NC: 5’-CCGGTTCTCCGAACGTGTCACGTTTCAAGAGAACGTGACACGTTCGGAGAATTTTTTG-3’.

### Quantitative real-time PCR

Total RNA was extracted using TRIzol reagent (Invitrogen, Boston, MA) and cDNA was synthesized with the Hifair III cDNA Synthesis Kit (Yeasen, 11141ES10, Shanghai, China). Real-time PCR was carried out with the Hieff UNICON qPCR SYBR Green Master Mix Kit (Yeasen, 11199ES03, Shanghai, China) on an Applied Biosystems 7500 Fast Real-Time PCR System. Relative mRNA expression was determined using the 2^-ΔΔCt^ method. Primers used are as follows: UNC5B (Human)-forward: CACGGGCGAGTCCTATTC; UNC5B (Human)-reverse: CGGCTCCTCCACCAAGTA; GAPDH (Human)-forward: ACAACTTTGGTATCGTGGAAGG; GAPDH (Human)-reverse: GCCATCACGCCACAGTTTC.

### Western blot

Total protein was extracted using RIPA buffer (Boster, Pleasanton, CA) supplemented with a protease and phosphatase inhibitor cocktail (Thermo Fisher Scientific, Boston, MA) and quantified using the BCA Protein Assay (Thermo Fisher Scientific, Boston, MA) according to manufacturer’s instructions. Protein lysates were separated using 10% SDS-PAGE and transferred to PVDF membranes. Subsequently, membranes were blocked with 5% non-fat milk and incubated with UNC5B (Bioss, 11492R, 1:1000, Woburn, MA), phospho-PI3K p85α (phospho Y607) (Abcam, ab182651, 1:1000, Cambridge, MA), PI3K p85α (Abcam, ab191606, 1:1000, Cambridge, MA), phospho-Akt (Ser473) (Cell Signaling Technology, 4060S, 1:2000, Danvers, MA), Akt (Cell Signaling Technology, 2920S, 1:2000, Danvers, MA), and GAPDH (Boster, BM1985, 1:2000, Pleasanton, CA) at 4° C overnight, followed by a one-hour incubation with secondary antibodies. The signal was detected using an enhanced chemiluminescence detection kit (Boster, Pleasanton, CA). Relative protein expression was measured with ImageJ and normalized over GAPDH.

### Tissue microarray and immunohistochemistry

A tissue microarray (TMA) containing 160 breast cancer tissues and 10 normal breast tissues was purchased from Outdo Biotech (Shanghai, China). The slide was deparaffinized and rehydrated before antigen retrieval. After endogenous peroxidase activity was blocked, the slide was blocked with 10% normal goat serum and incubated with a UNC5B polyclonal antibody (Thermo Fisher Scientific, PA5-67631, 1:25, Boston, MA) at 4 °C overnight. The slide was then incubated with secondary antibody, stained with diaminobenzidine, and counterstained with hematoxylin.

The UNC5B expression was determined by two pathologists according to the immunoreactivity score (IRS). The immunostaining intensity (negative, 0; weak, 1+; moderate, 2+; strong, 3+) and the percentage of positive cells (0-5%, 0; 6%-25%, 1+; 26%-50%, 2+; 51%-75%, 3+; 76%-100%, 4+) were recorded, and the IRS was calculated by multiplying the two scores. Because four tumor cores were lost during IHC staining, 156 tumor cores were analyzed, and tumor samples were classified into two groups, high expression and low expression, by their median expression. *p* values were generated by the log-rank test with *p* < 0.05 considered statistically significant.

### GEPIA analysis

The GEPIA (http://gepia.cancer-pku.cn/index.html) is a web-based tool for diverse gene expression analysis based on 9,736 tumors and 8,587 normal samples from the TCGA and the GTEx databases [40]. Here, we used the *Expression* module to analyze the mRNA expression of UNC5H receptors in breast cancer and normal breast tissues. We also used the *Survival* module to evaluate the prognostic value of UNC5B in breast cancer. Breast cancer samples were separated into two groups, high expression and low expression, by their median expression, and *p* values were determined by the log-rank test.

### cBioPortal analysis

The cBioPortal for Cancer Genomics (http://cbioportal.org) is a user-friendly web resource that provides access to multiple cancer genomic data sets [41]. We explored the metastatic breast cancer project (Provisional, October 2018) dataset and analyzed genomic alterations of UNC5B, which contained mutations and putative copy-number alterations from GISTIC. The *OncoPrint* module provided an overview of the genomic profile in UNC5B, and the *Network* module was used to visualize biological interactions between UNC5B and the 20 most frequently altered neighboring genes in breast cancer. GO and KEGG pathway enrichment analyses of altered neighboring genes were conducted with the “clusterProfiler” package in R.

### Cell proliferation assay

Breast cancer cell proliferation was determined using a CCK-8 kit (Dojindo, CK04, Tokyo, Japan). Briefly, 2,500 cells/well were seeded into a 96-well plate and incubated at 37 °C. After culturing for 24-96 h, cells were incubated with CCK-8 reagent according to manufacturer’s instructions and absorbance at 450 nm was measured.

### Cell colony formation assay

A total of 1,000 cells/well were seeded into a 6-well plate and incubated at 37 °C. After culturing for 2 weeks, colonies were fixed, stained with crystal violet, and counted. Experiments were repeated at least three times independently.

### Cell migration and invasion assay

Cell migration and invasion assays were performed using Transwell chambers (Corning, 3422, NYC), without Matrigel (Corning, 356230, NYC) for migration assays and with Matrigel for invasion assays. Briefly, T-47D cells (10 × 10^4^ cells/chamber) or MDA-MB-231 cells (5 × 10^4^ cells/chamber) in 200 μL of serum-free DMEM or L15 medium were seeded in the upper chambers and 500 μL of corresponding medium containing 10% FBS were placed in the lower chambers. After 24 h, cells on the lower surface of the membrane were fixed and stained with crystal violet. Cells were counted in five random fields under a microscope.

### LinkedOmics database

The LinkedOmics database (http://linkedomics.org/login.php) encompasses multi-omics data for 32 cancer types from the TCGA project and provides comprehensive analyses of more than a billion data points [42]. We used the *LinkFinder* module to analyze and visualize UNC5B-correlated genes that were differentially expressed in 1,093 breast cancer patients. Pearson’s correlation test was used to measure the strength of the correlations. Significantly co-expressed genes correlated with UNC5B were further extracted using the criterion of FDR<0.05, and GO and KEGG enrichment analyses were performed with the “clusterProfiler” package in R.

### Statistical analysis

Data were analyzed in GraphPad Prism 7 (GraphPad Software) and were presented as the mean ± s.e.m. Unless otherwise stated, statistical significance was analyzed by either two-tailed, Student’s t-test or one-way ANOVA. *p* values < 0.05 were considered statistically significant.

## Supplementary Material

Supplementary Figures

Supplementary Table 1
