# Supplementary material for: High expression of UNC5B enhances tumor proliferation, increases metastasis, and worsens prognosis in breast cancer
Source: Aging (Albany NY). 2020 Sep 9;12(17):17079–98. doi: 10.18632/aging.103639 (PMC7521535; doi:10.18632/aging.103639)
Supplement: Supplementary Figures [file aging-12-103639-s002..pdf]

SUPPLEMENTARY FIGURES

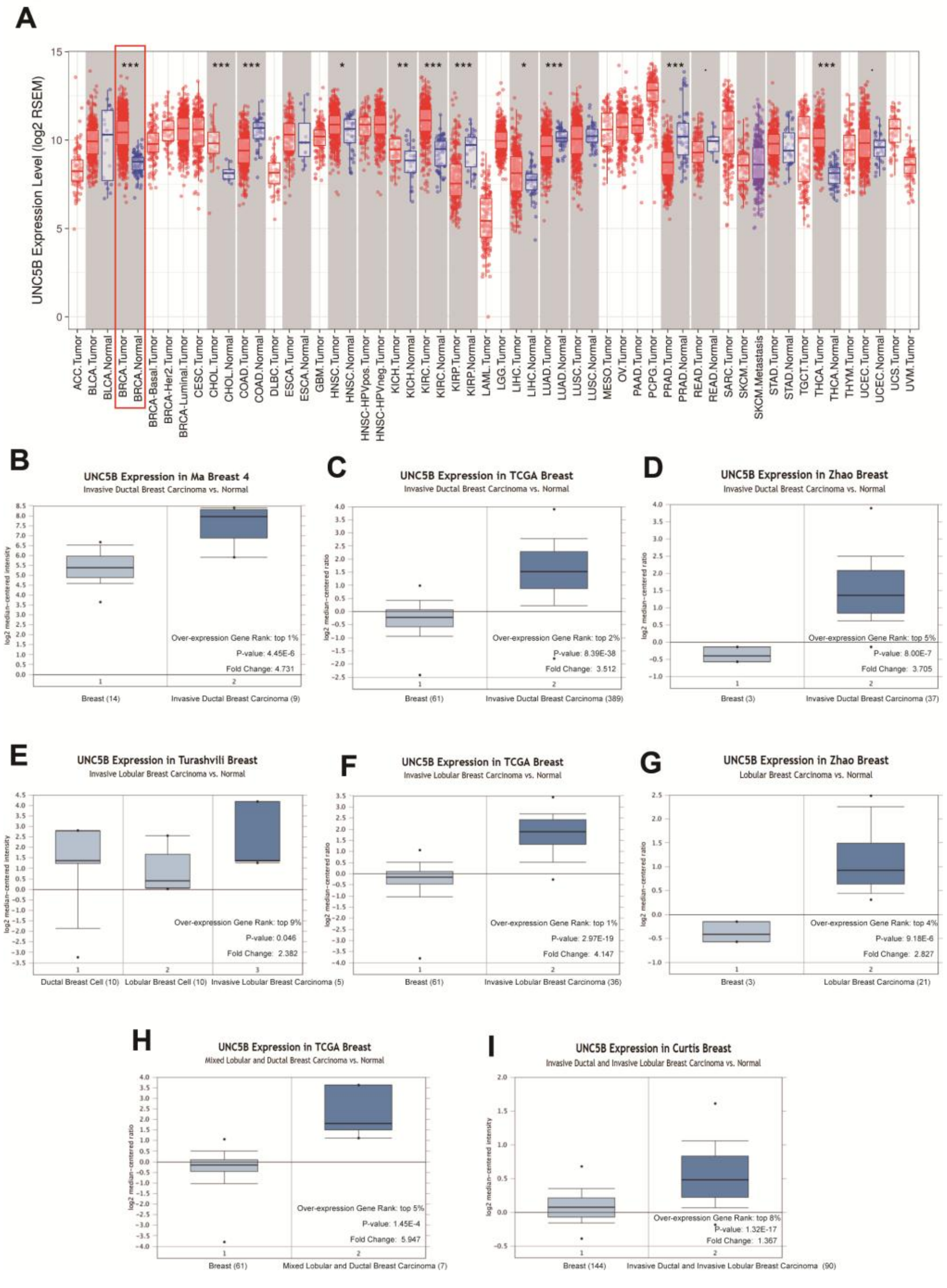

Supplementary Figure 1. UNC5B mRNA expression is upregulated in breast cancer (TIMER and ONCOMINE). (**A**) UNC5B mRNA expression levels between tumor tissues and adjacent normal tissues in 32 cancer types from TCGA. (**B**–**D**) UNC5B mRNA expression levels in invasive ductal breast carcinoma compared with normal breast tissues. (**E**–**G**) UNC5B mRNA expression levels in lobular breast carcinoma compared with normal breast tissues. (**H**, **I**) UNC5B mRNA expression levels in mixed lobular and ductal breast carcinoma compared with normal breast tissues. Data shown in Figure S1B-I are over-expression gene rank, related *p* - value, and fold change generated by Oncomine. *, $p < 0.05$; **, $p < 0.01$; ***, $p < 0.001$.

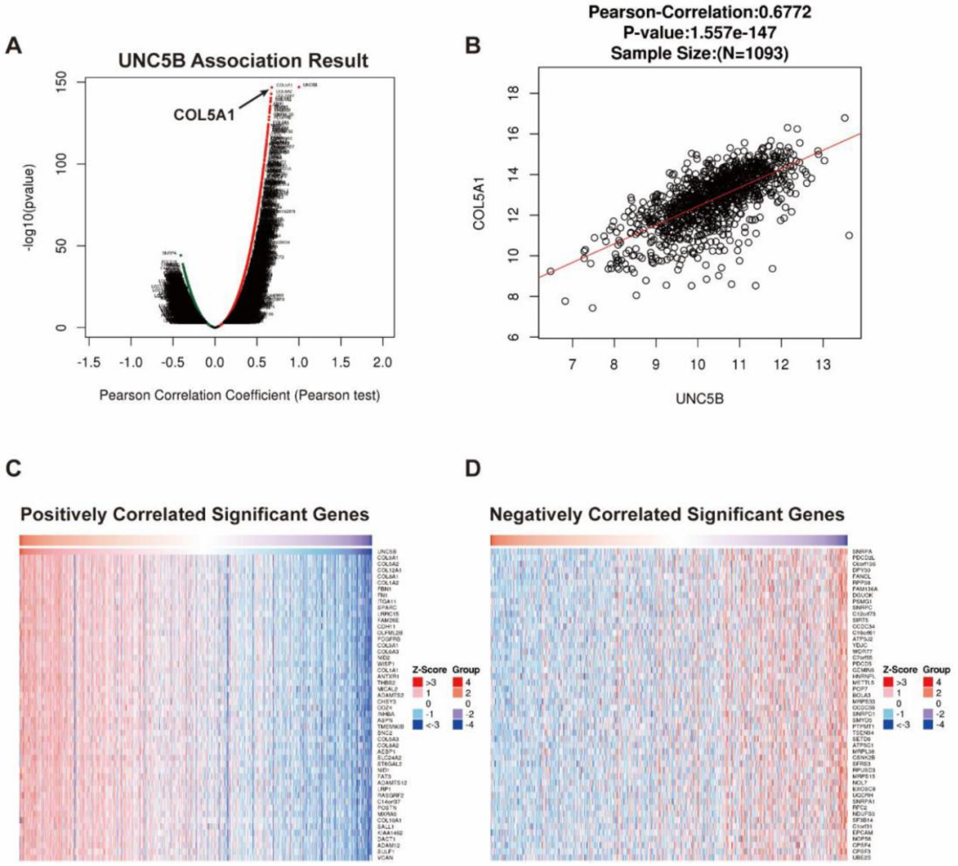

Supplementary Figure 2. Differentially expressed genes associated with UNC5B in breast cancer (LinkedOmics). (**A**) Volcano plot showing the relation of UNC5B to differentially expressed genes in breast cancer. (**B**) Pearson correlation between the expression of UNC5B and COL5A1 in breast cancer. (**C**, **D**) Heat maps showing the top 50 genes positively and negatively correlated with UNC5B in breast cancer.
