# Supplementary material for: High expression of UNC5B enhances tumor proliferation, increases metastasis, and worsens prognosis in breast cancer
Source: Aging (Albany NY). 2020 Sep 9;12(17):17079–98. doi: 10.18632/aging.103639 (PMC7521535; doi:10.18632/aging.103639)
Supplement: Supplementary Table 1 [file aging-12-103639-s001..pdf]

SUPPLEMENTARY TABLE

Supplementary Table 1. The characteristics of UNC5B-neighboring gene alterations in breast cancer (cBioPortal).

Gene Symbol	Amplification	Mutation	Homozygous Deletion	Total Alteration
UNC5B	10.5	0.8	0	11.0
DAPK1	4.6	0.8	0	5.5
DAPK3	0	0	7.6	7.6
FRS2	4.2	0	0	4.2
FYN	4.6	0	0.8	5.5
GRB2	8.4	0	0.4	8.9
KIRREL1	5.9	1.7	1.7	9.3
NPHS1	2.5	1.3	10.5	14.3
NPHS2	9.7	1.3	0	10.5
NTN3	1.3	0	4.6	5.9
NTN4	4.6	0.4	0	5.1
NTNG1	2.5	0.8	0.4	3.8
PIK3CA	2.5	32.1	0	32.5
PIK3CD	0	0.8	1.7	2.5
PIK3CG	0.4	1.3	0.8	2.5
PIK3R1	7.2	1.7	0.8	9.3
RAC1	3.4	0	0.8	4.2
RAC2	0	0	3	3
RHOU	11	0	0	11
SDC2	12.7	0.4	0	13.1
UNC5A	1.3	0	1.3	2.5
